# Mylohyoid Ridge as a Predictor of Available Bone for Implant Placement: A Cone-Beam Computed Tomography (CBCT) Retrospective Observational Study

**DOI:** 10.7759/cureus.27470

**Published:** 2022-07-29

**Authors:** Sakshi Madhok, S Kiruthika, K Prabhu, Sonia Abraham, P Kabilan, S Nithyapriya

**Affiliations:** 1 Prosthodontics and Crown and Bridge, Adhiparasakthi Dental College and Hospital, Melmaruvathur, IND

**Keywords:** internal oblique ridge, mandibular canal, mylohyoid ridge, inferior alveolar canal, submandibular fossa

## Abstract

Introduction: The posterior mandibular region, due to the presence of vital structures, poses a high risk during implant placement because of its susceptibility to neurovascular injury and perforation of the lingual cortex. A breach in implant length and available bone height may lead to serious intraoperative and postoperative complications. Prediction of the exact location of the inferior alveolar nerve and submandibular fossa anatomy is a prerequisite for ideal implant placement, which is always not possible with conventional radiographic and clinical techniques.

Materials and methods: One hundred ten cone-beam computed tomographies (CBCTs) of patients were acquired from the radiological archives of a radiological center in Chennai. DICOM files from CBCT were exported to Bly Sky Plan software. Cross-sections of the second molar and first molar were extracted following the inclusion criteria. The linear dimension between the mandibular canal and mylohyoid ridge and anatomic variables of the submandibular fossa were measured digitally on the left and right sides using software measuring tools. Descriptive statistics were done. The unilateral and bilateral site and gender differences were evaluated. Bone height superior to the mandibular canal was correlated with the submandibular fossa parameters; depth of undercut in the vertical and horizontal directions; and angle of the undercut.

Results: The mandibular canal was on average 5.5 mm and 4 mm inferior to the Mylohyoid ridge in the second molar region and first molar region, respectively, with the right and left sides showing no statistically significant difference. The depth of fossa undercut in vertical and horizontal dimensions was higher in the second molar region compared to the first molar region. The height of the deepest point of the undercut in the vertical dimensions showed a positive correlation with the bone available between the mandibular canal and the mylohyoid ridge.

Conclusion: Keeping 2 mm of safety factor in consideration, implants can be safely placed up to the mylohyoid ridge in 100% of cases and 2 mm below the mylohyoid ridge in 78.9% of cases in the mandibular second molar region. In keeping with a safety factor of 2 mm, implants can be safely placed up to the mylohyoid ridge in 82.6% of cases and 2 mm below the mylohyoid ridge in 43.1% of cases in the first molar region. A more pronounced undercut was seen in the second molar region than in the first molar region. Deeper fossa undercuts in vertical dimension are associated with more inferior positioning of the mandibular canal.

## Introduction

The posterior mandible poses a significant implant surgical risk [1]. In the absence of proper preoperative assessment of implant length and angulation, this region is susceptible to neurovascular injury and perforation of the lingual cortex [2,3]. Important determinants of implant placement in the posterior mandible are the submandibular fossa (SMF) and the mandibular canal (MC), which show variability that restricts ideal implant placement. The first characteristic to be evaluated, according to Froum et al., should be the location of the MC, followed by the risk of perforation on the SMF [4]. Management of submandibular undercut involves placement of a buccally angulated implant, a short implant [2,5], or horizontal augmentation of SMF [6]. The position of the MC allows quantification of implant length. History is replete with reported cases of lingual cortical perforation [7-10] and inferior alveolar nerve injury [11,12] during implant surgery. Thus, there was a need to have added vital information about this critical zone and to devise an alternative landmark/method that could act as a guide to assess the vertical height of bone in the posterior mandible.

Mylohyoid ridge (MR) is an important anatomic structure to be considered while rendering prosthetic treatment [13-16] but is seldom described as a potential anatomic guide to determine the available bone height in the mandibular posterior region. MC is a non-palpable radiographic landmark. In contrast, MR, also known as the internal oblique ridge, is a palpable anatomic [16] and radiographic landmark [17]. The internal MR onto which the mylohyoid muscle attaches and the SMF are inherently considered to be non-resorbable structures as they are integral parts of the basal bone of the mandible [18-20]. Loss of premolars and molars has no effect on MR position [21], but MR prominence increases with continuous resorption and period of edentulousness [16]. Palpation of the MR and SMF is traditionally used to subjectively assess the depression in the mandible's posterior lingual cortex [1,22]. The line is well evident in patients' casts [23]. The relative position of the MR with respect to the body of the mandible has been discussed [24]. Studies have determined the relative position of the inferior alveolar canal with respect to various anatomic landmarks like the inferior border of the mandible [2], root apices of mandibular posterior teeth [25,26], ridge crest and external mandibular cortex [27]. However, no study has addressed the relative position of the MC with respect to the MR.

Clinicians usually palpate this anatomically complex mandibular posterior region before implant placement, which depends on their subjective perception as well as the patient-to-patient anatomic variability of SMF [5,28]. Many studies have cross-sectionally evaluated the dimensions and morphology of SMF and visualisation of the MC in the posterior mandible and emphasised the significance of submandibular undercut and resultant achievable implant angulation [2,17,29]. These studies have emphasised the significance of morphological and anatomic variegation of the fossa in isolation. However, De Souza et al have correlated the horizontal and vertical bone dimensions in the posterior mandible with the horizontal SMF depth and age [30]. Changes in the shape of the MC have also been linked to the shape of the face [31].

It is vital to have additional information regarding the intricate anatomy of the submandibular region, a potential risk in implant surgery. This could better assist clinicians in implant planning decisions. So, a study was set up to look at the straight line between the MC and the MR and see if there was a link with the morphologic variables of SMF. The null hypothesis is that there is no linear relationship between the MR and the MC in the M1 and M2 regions. These findings can be used to project the average superio-inferior position of the MC, derive an anatomic average between the MC and the MR, and provide additional important information about implant length and angulation during the implant planning stage. The goals of the study were to find out the average distance between MC and MR in the mandibular M1 and M2 region on both sides, find out the morphologic variables of SMF undercut in terms of depth in the horizontal dimension, relative depth in the horizontal dimension, depth in the vertical dimension, angle, and relative angle, and find a relationship between the bone height between MC and MR and the anatomical variables of SMF.

## Materials and methods

The study was approved by the Institutional Review Board of Adhiparasakthi Dental College and Hospital (2020-IRB-Mar-Prosth01/APDCH). This retrospective observational/exploratory study was performed using cone-beam computed tomography (CBCT) scans of patients. The sample size was collected by imitating previous similar studies [2] and the convenience of data collection. One hundred ten CBCT data of Indian patients were retrieved randomly from the radiological archives of a radiological center in Chennai. These patients were advised CBCT by private clinicians for diagnosis of surgical lesions or endodontic treatment planning. No patient was exposed to CBCT radiation, especially for the purpose of the study. The field of view was 16х16 as this was the most readily available scan in the archive. CBCT datasets were transferred to a laptop installed with Blue Sky Plan software version 4.5.9 (Blue Sky Bio, New York City, United States). The anonymity of the patients and confidentiality of the data were ensured. All CBCTs were taken from the same machine; the Plameca Promax CBCT machine that utilizes Romexis software by single trained personnel following manufacturer-recommended protocol and settings. The imaging parameters were set at 120kvp, 18.66 mAs, and scan time-20 seconds with a resolution of.4mm.

**Figure 1:**
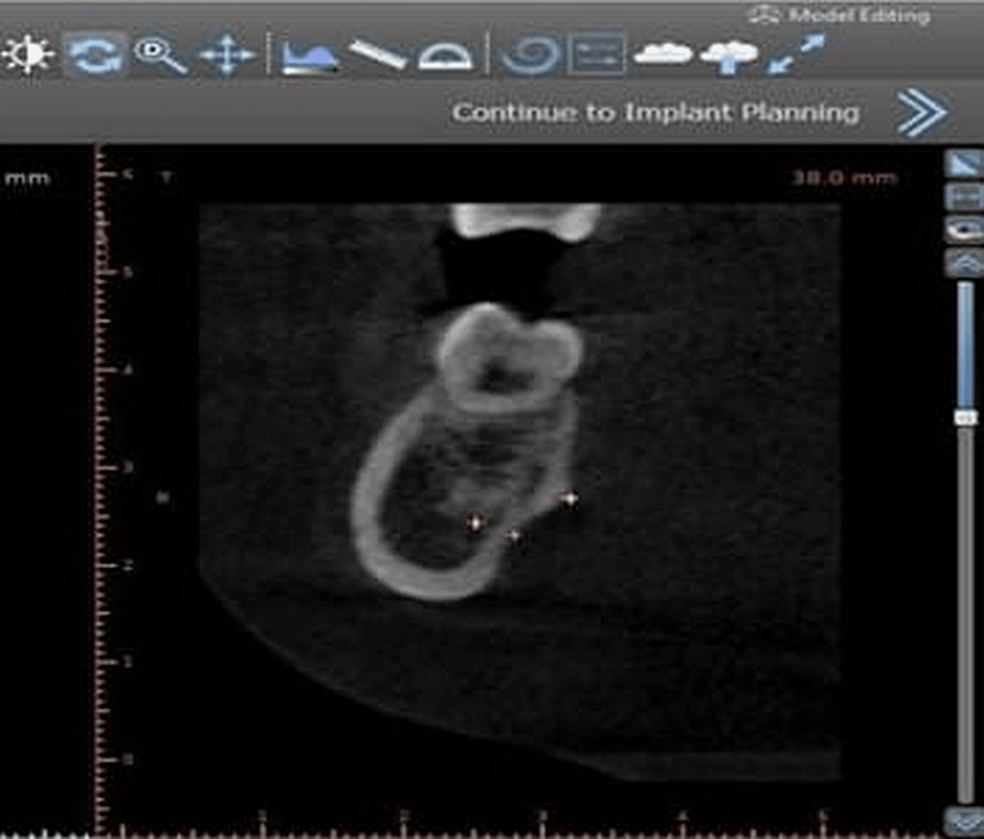
CBCT with three reference point

Digital imaging and communications in medicine (DICOM) files were retrieved from CBCT datasets and imported into Blue Sky Plan software program. Reformatting of files was done to obtain transverse cross-sectional images. The jaw was traced on the axial section using a curvilinear reformatting tool. The transverse cross-sections were then automatically generated by the program and screened for reference point identification (Figure 1). Default image section thickness was taken, i.e., 1mm. 

**Figure 2:**
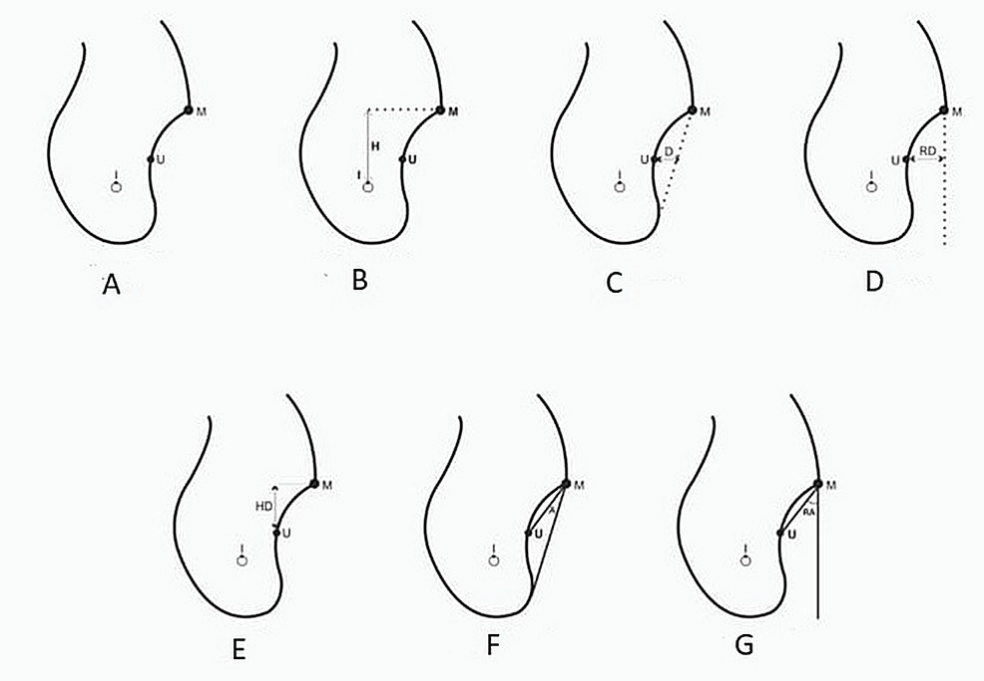
Diagramatic representation of reference point

One hundred ten artifact-free scans of Indian patients aged 18 years or more that had a full complement of mandibular posterior teeth excluding mandibular third molars with absence of radiological evidence of skeletal, dental malocclusion and periodontitis or drifted teeth were randomly selected initially. We thus had 220 hemi mandibles from CBCT of 110 patients. Two cross sections were screened through each hemimandible in the software, each passing through the furcation of first molar (M1) and second molar (M2). Three reference points were marked on each cross section (Figure 2A).

Point I - Point at the height of MC cortication. Point M - Most prominent point on the lingual surface (at the height of undercut) representing MR [17]. Point U - Deepest point in the undercut. Any discrepancy in discrete identification of reference points qualified the section to be further excluded from the sampling. Therefore, scans with nonidentifiable MC, indistinct prominent MR, absence of submandibular undercuts, and presence of posterior pathologies were excluded. Oral and maxillofacial radiologist was responsible for qualifying the cross sections as a sample and identifying and labelling the reference points in cross sections. This left us finally with 67 cross sections for right second molar, 62 for right first molar, 49 for right first molar and 60 for left first molar.

Following this, linear and angular measurements were made from each qualified cross section. Default digital linear and angular measuring tool of blue-Sky Bio software was used for recording measurements and angulations. All the readings were recorded by the same radiologist twice. The mean of the values was recorded as the final linear and angular measurement of variables. H representing distance between superior surface canal cortication and MR and was measured by recording the shortest distance between point I and point M (Figure 2B). D represents the depth of undercut in horizontal direction; distance between point U and a line joining point M and the inferior most prominent point on the lower border of the mandible (Figure 2C). RD represents the relative depth of the undercut in horizontal direction measured by the distance between point U and tangent dropped perpendicular from point M (Figure 2D). HD represents height of the deepest point of the undercut from MR (in vertical direction); measured by recording the shortest distance between point U and point M (Figure 2E). A represents the angle of the undercut; recorded by angle formed between line joining point U with point M and point M with the inferior most prominent point on the lower border of mandible (Figure 2F). RA represents the relative angle of the undercut; measured by the angle formed by line joining point U with point M and a tangent dropped perpendicular from point M (Figure 2G).

The study population ranged from 18 to 64 years with an average age of 43.2. The data obtained were subjected to statistical analysis using Statistical Package for the Social Sciences (SPSS) Version 26.0. P-value<0.05 was considered to be statistically significant.

## Results

The normality tests, Kolmogorov-Smirnov and Shapiro-Wilks tests results reveal the study followed a normal distribution. Therefore, to analyze the data, parametric tests were applied. Table 1 presents the descriptive statistics of bone height between MC and MR (H) and SMF parameters.

**Table 1:** Descriptive statistics Right Second Molar-RM2, Left Second Molar-LM2, Right first Molar-RM1, Left first Molar-LM1, H: Bone height, HD: deepest point of undercut, D: depth of undercut in the horizontal direction, A: angle of undercut, RA: relative angle of undercut, RD: relative depth of undercut in the horizontal direction, SD: standard deviation

VARIABLES	RM2		LM2		RM1		LM1	
	MEAN	SD	MEAN	SD	MEAN	SD	MEAN	SD
H (mm)	5.41	2.47	5.65	2.54	4.01	2.15	3.97	2.04
D (mm)	1.85	0.68	2.03	0.70	1.41	0.53	1.57	0.51
HD (mm)	5.35	1.56	5.85	1.49	4.34	1.61	4.39	1.15
RD (mm)	4.28	1.29	4.87	1.15	2.99	1.09	3.21	0.98
A (degree)	12.97	5.31	12.47	5.53	14.3	9.92	14.05	7.05
RA (degree)	37.94	9.76	38.73	9.24	33.5	10.88	35.50	9.82

The mean distance between MC and MR in (right molar) RM2 is 5.41+2.47, (left molar) LM2 is 5.65+2.54, RM1 is 4.01+2.15 and LM1 is 3.97+2.04. Unpaired t-test is done to assess the mean gender (Tables 2, 3) and site (Table 4) difference.

**Table 2:** Gender difference in means of RM2 and LM2 *: statistically significant Right Second Molar-RM2, Left Second Molar-LM2, Right first Molar-RM1, Left first Molar-LM1, H: Bone height, HD: deepest point of undercut, D: depth of undercut in the horizontal direction, A: angle of undercut, RA: relative angle of undercut, RD: relative depth of undercut in the horizontal direction, SD: standard deviation

No	Variable	Range	Gender	Number	Mean	Standard deviation	P-value
RM2	H	1.18-12.96	M	34	4.9215	2.03825	0.97
			F	33	5.8682	2.82002	
	D	0.78-3.61	M	34	2.0506	0.76741	0.010*
			F	33	1.6261	0.48717	
	HD	1.78-8.40	M	34	5.4806	1.38134	0.53
			F	33	5.2697	1.74622	
	RD	2.00-7.09	M	34	4.6982	1.41494	0.001*
			F	33	3.8791	1.04143	
	A	1.25-24.52	M	34	13.5873	5.51201	0.226
			F	33	12.0288	4.76115	
	RA	16.94-64.09	M	34	39.7894	10.03815	0.07
			F	33	35.7945	9.19189	
LM2	H	2.26- 10.19	M	35	4.9370	1.94746	0.019 *
			F	27	6.5581	2.98938	
	D	0.85-3.74	M	35	2.1596	0.80002	0.77
			F	27	1.8444	0.49038	
	HD	3.58-7.69	M	35	5.8611	1.26912	0.815
			F	27	5.9626	1.76641	
	RD	2.28-7.51	M	35	5.0907	1.38009	0.128
			F	27	4.5459	0.87083	
	A	1.31-27.8	M	35	12.2652	5.78267	0.958
			F	27	12.1963	4.68250	
	RA	15.9-57.26	M	35	38.6626	9.25729	0.594
			F	27	37.4307	9.19720	

**Table 3:** Gender difference in means of RM1 and LM1 *: statistically significant Right Second Molar-RM2, Left Second Molar-LM2, Right first Molar-RM1, Left first Molar-LM1, H: Bone height, HD: deepest point of undercut, D: depth of undercut in the horizontal direction, A: angle of undercut, RA: relative angle of undercut, RD: relative depth of undercut in horizontal direction, SD: standard deviation

No	Variable	Range	Gender	Number	Mean	Standard deviation	P-value
RM1	H	2.26- 10.19	M	25	3.4458	2.02884	0.126
			F	24	4.4879	2.19518	
	D	0.56-2.72	M	25	1.5388	0.55964	0.08
			F	24	1.2529	0.46898	
	HD	2.53-7.81	M	25	4.6550	1.46879	0.207
			F	24	4.0596	1.75495	
	RD	1.79-5.29	M	25	3.4092	0.90618	0.013*
			F	24	2.5712	1.14129	
	A	1.46-61.62	M	25	15.5587	12.41710	0.319
			F	24	12.7183	6.67269	
	RA	14.70-49.90	M	25	34.7538	9.17335	0.426
			F	24	31.9271	12.47288	
LM2	H	1.20-9.31	M	33	3.2974	1.60707	0.021*
			F	27	4.5800	2.22072	
	D	0.95-3.39	M	33	1.5804	0.53079	0.772
			F	27	1.5374	0.54972	
	HD	2.42-6.13	M	33	4.3737	1.30664	0.628
			F	27	4.2307	0.85107	
	RD	1.28-4.88	M	33	3.2052	0.89124	0.322
			F	27	3.1048	0.95991	
	A	1.71-35.00	M	33	14.2570	7.92479	0.944
			F	27	14.2741	6.77809	
	RA	14.43-55.50	M	33	35.5907	10.45302	0.954
			F	27	35.7722	9.76251	

**Table 4:** Unilateral and bilateral comparison of means *: statistically significant Right Second Molar-RM2, Left Second Molar-LM2, Right first Molar-RM1, Left first Molar-LM1, H: Bone height, HD: deepest point of undercut, D: depth of undercut in horizontal direction, A: angle of undercut, RA: relative angle of undercut, RD: relative depth of undercut in horizontal direction, SD: standard deviation

VARIABLES	P-Value			
	R 2^ND ^& L 2^ND^	R 1^ST^ & L 1^ST^	R 2^ND^ & R 1^ST^	L 2^ND^ & L 1^ST^
H	0.699	0.456	0.001	0.001*
D	0.05	0.150	0.004	0.001*
HD	0.051	0.794	0.006	0.001*
RD	0.002	0.425	0.001	<0.01*
A	0.610	0.989	0.511	0.129
RA	0.398	0.486	0.076	0.104

Minimal site and gender differences were observed. Correlation was assessed bilaterally using Pearson correlation test (Table 5).

**Table 5:** Pearson correlation between H and variables of submandibular fossa *: statistically significant Right Second Molar-RM2, Left Second Molar-LM2, Right first Molar-RM1, Left first Molar-LM1, H: Bone height, HD: deepest point of undercut, D: depth of undercut in the horizontal direction, A: angle of undercut, RA: relative angle of undercut, RD: relative depth of undercut in horizontal direction, SD: standard deviation, sig: significant

VARIABLES			D	HD	RD	A	RA
RM2	H	Pearson Correlation	0.046	0.400	0.116	-0.020	0.389
		Sig. (2-tailed)	0.709	0.001*	0.351	0.872	0.001*
		N	67	67	67	67	67
		LM2		Pearson Correlation	0.148	0.360	0.014	0.120	0.235
Sig. (2-tailed)	0.251			0.004*	0.914	0.354	0.066
N	62			62	62	62	62
RM1		Pearson Correlation	0.144	0.334^*^	-0.036	0.116	0.245
		Sig. (2-tailed)	0.324	0.019*	0.808	0.426	0.090
		N	49	49	49	49	49
LM1		Pearson Correlation	0.183	0.246	0.027	0.029	0.166
		Sig. (2-tailed)	0.161	0.058	0.857	0.827	0.205
		N	60	60	60	60	60

Bone height (H) has a positive correlation with height of deepest point of undercut (HD) bilaterally in M2 region. The same variables demonstrated a positive correlation in RM1 but not in RM2.

## Discussion

The mandibular posterior region is highly susceptible to surgical trauma and mishaps due to high vascularization, presence of vital structures (inferior alveolar nerve, muscle attachments, submandibular gland), undercut and a varying amount of bony atrophy. Thus, this region demands meticulous preoperative planning and execution [2]. Conventionally this susceptible zone is assessed by means of palpation, bone calipers, flap elevation with direct viewing, and evaluation of dental cast models. Our study aimed at relating MR and MC to provide an anatomic average and correlating this value with the variables of SMF. This knowledge will help in identifying a certain type of bone and SMF morphology that can simplify implant planning and make dental imaging more selective and efficacious.

This study could have been performed in three ways. First is by sectioning the dry skull and measuring the dimensions. This method was not used because of the disadvantages related to shrinkage caused by dry skull and fracture of subtle dehydrated brittle structures during sectioning of dry cadaver skulls. Second by doing CBCT of cadaveric skulls. Though CBCT allows accurate image reproduction, reliable visualisation of MC, and measurements of available bone [32-34], this method was not preferred because of practical difficulty in translating the cadaveric findings to population owing to differences in identification of age, disease, and sex. Considering CBCT to be the best non-invasive method [2,5] for bone dimension assessment and implant planning, CBCT of live patients was used in this study. We achieved more number of cross sections from M2 than M1. This could be because of the easier identification of MC cortication in posterior sections than in anterior sections [32].

In our study, the mean vertical height of available bone between MC and MR was found to be 5.4 mm and 5.6 mm in RM2 and LM2, respectively. In M1, the canal was 4.01 mm inferior to MR on the right side and 3.97 mm on the left side. No statistically significant difference was seen on the right and left sides. This mean value added to the residual alveolar bone available at the time of implant placement projects the bone height available for implant placement in the mandibular posterior region. These averages can help us in assessing the position of MC in the superio-inferior plane upon digital palpation or visual location of the MR during flap reflection. Denio et al. did a cadaveric study to assess the mean distance between MC and M2 and M1. They found the values to be 3.7 mm and 6.9 mm, respectively [35]. On the other hand, our study showed a 5.5 and 4 mm distance between MC and MR in M2 and M1 regions. This can perhaps be explained by a superior position of MR with respect to the root apices in the M2 region. In addition, a study assessing the positional relation of the MR to root apices of MC stated that the root apex of M2 is located below the MR [24]. This difference could also be due to racial and ethnic anatomic differences between the study population. Further studies are needed in the future to prove this hypothesis. Littner et al. reported the upper border of the MC was located 3.5-5.4 mm below the root apices of M1 and M2 [36]. The amount of bone present in M2 was statistically higher than in the M1 region. This shows more amount of bone in the M2 region compared to the M1 region when MR is taken as the reference.

Average lingual concavity depth in the horizontal direction was found to be 4.28, 4.87, 2.99, and 9.63 in RM2, LM2, RM1, and LM1, respectively. No statistically significant difference was seen on the right and left sides, but statistically significant pronounced undercuts were seen in the M2 region compared to the M1 region. Parnia et al. said that SMF depth greater than 2 mm is a potential risk factor for lingual cortex perforation during implant placement [5]. A lingual undercut greater than 2 mm was found in 80% of his samples. Our 100% samples show an undercut value of more than 2 mm. Various mean depths of undercut have been reported. One study has reported a mean depth of lingual undercut as 3.7 mm [37]. Salemi et al. in a CBCT study assessed the lingual undercut in the first molar edentulous site and gave a range of undercut depths ranging from 7 to 4.9 mm [1]. Kamburoglu et al. have reported a mean depth of 1.3 mm [38], those are the references of the articles that have included all convex, concave and parallel cross sections in their research paper, unlike our study where only undercut cross sections are included. Our values are higher and different than the previously quoted values because of including only the undercut type of cross sections and excluding the parallel and convex cross-sections from the sampling [1,2,37]. This information is derived from the methodology used previously which has used all parallel, undercut and convex cross sections in their methodology to calculate the mean. Whereas our study design included only undercut type of cross sections, and this could be one of the reasons our mean value was higher than other studies. As precise identification of MR is possible only in a section with a prominent undercut. In addition, differences in means of variables may also be due to the use of different reference points, racial and ethnic variation, assessing a dentulous or edentulous site, etc. We have included patients with a full complement of teeth in our study to ensure accurate identification and localisation of mid-molar sections which was not possible by the methodology followed by other authors [30].

The height of the deepest part of the undercut is 5.35, 5.85, 4.34, and 4.39 in RM2, LM2, RM1, and LM1 regions, respectively. This shows vertically deeper undercuts in M2 compared to M1 which is also statistically significant. No statistically significant difference is seen in bilateral sites. This is in unison with other studies that state that more pronounced undercuts are seen in the M2 region compared to the M1 region [17].

Correlation between supracanal bone height versus the SMF parameters showed a significant positive correlation between H and HD in the second molar region (r - 0.4 RM2 and 0.36 LM2). A statistically significant positive correlation was also seen between supracanal bone height and depth of undercut in the vertical axis in the RM1 (r - 0.334) region. The positive correlation indicates deeper the undercut in the vertical dimension is more inferior to the position of MC with regard to MR. Thus, while evaluating a ridge digitally or visually on flap reflection, a more pronounced undercut in the vertical dimension is associated with more inferiorly positioned canals and safer placement of implants in the second molar region. In a similar study, it was demonstrated, that a positive correlation existed between buccolingual bone width and SMF depth in the horizontal dimension [30]. They also correlated the bone height (Alveolar crest to MC) with a depth of fossa in the horizontal dimension and did not find any significant correlation between the two.

It is recommended that two-dimensional imaging be the first assessment radiograph for implant planning [39]. The SMF cannot be visualized in any 2D imaging modality. 28% of periapical radiographs do not reveal the distinct position of inferior MC in the mandible [35]. Therefore, if the MC is not visible in the periapical film, it is recommended to obtain the panoramic image. Only 36.7% of panoramic radiographs reveal distinct MC [40]. If still the canal cannot be located; a 3D imaging modality is recommended. 2% of CT scans and 60% of complex tomography as well fail to reveal the MC [41]. Also, concern has been raised regarding the potential radiation risk associated with CT [42]. A comparison of the accuracy of periapical, panoramic and CT images in locating the MC has shown a mean linear radiographic error of 14%, 23% and 1.8%, respectively [43]. Accurate cross-sectional imaging is indispensable and an invaluable guide for confirming the location of MC. It was found in a study that MC is clearly visible only in 53% of CBCT [32]. Further, commonly clinicians especially in developing countries choose to proceed with implant placement without CBCT due to either non-accessibility to 3D imaging or unaffordability by the patients. Results of the study can be clinically applied for pre-implant assessment of the bone. It is already proved that MC lies below the apex of the mandibular posterior root tips. Our study also shows that MC is on average about 4-5.5mm below the mylohyoid line in the mandibular posterior M1 and M2 regions, respectively. This is important information for pre-radiographic implant site assessment for bone availability in posterior mandibular regions. In such a scenario the knowledge of our study can prove to be vital. Various techniques have been historically used to assess the morphology of the implant site before implant placements. These include ridge and SMF palpation, cast analysis, use of osteometre, etc., but all with their limitations [5]. The inference drawn from our study could be a new addition to the range of techniques to assess the morphology and bone dimension of the mandibular posterior implant site before implant placement. As there is no significant difference in the value of H on the right and left sides our study shows that MC is 5.5 mm below the MR in the second molar region and 4 mm below the MR in the first molar region. All the cross sections have shown that MC is inferior to the ridge also called the mylohyoid line in M1 and M2 region. Respecting a safety margin of 2 mm [44,45], it is interpreted from this study that implants can be safely placed up to the MR in 100% of cases and 78.9% of times 2 mm below the MR in the M2 region. Similarly, in the M1 region implants can be safely placed up to MR in 82.6% of cases and 2 mm below the MR in 43.1% of cases. However, these findings are only applicable to sites with submandibular undercut which can either be palpated or visually appreciated on flap reflection. The result of this study is also not applicable in rare cases of extreme pathogenic resorption. Further large-scale studies with a robust study design on varying populations assessing age-wise and gender-wise associations are required to substantiate the findings of our study.

## Conclusions

The study gives a range of linear dimension depicting the height of bone between MC and MR, the depth and distance of SMF undercut from the MR. This information is quite helpful in preoperatively assessing the available bone above MC and invaluable in prevention of unwanted complications during mandibular posterior implant surgery.
